# Risk Factors for Vascular Occlusive Events and Death Due to Bleeding in Trauma Patients; an Analysis of the CRASH-2 Cohort

**DOI:** 10.1371/journal.pone.0050603

**Published:** 2012-12-10

**Authors:** Louise Pealing, Pablo Perel, David Prieto-Merino, Ian Roberts, CRASH-2 Trial Collaborators

**Affiliations:** Department of Population Health, London School of Hygiene and Tropical Medicine, London, United Kingdom; Leiden University Medical Center, The Netherlands

## Abstract

**Background:**

Vascular occlusive events can complicate recovery following trauma. We examined risk factors for venous and arterial vascular occlusive events in trauma patients and the extent to which the risk of vascular occlusive events varies with the severity of bleeding.

**Methods and Findings:**

We conducted a cohort analysis using data from a large international, double-blind, randomised, placebo-controlled trial (The CRASH-2 trial) [1]. We studied the association between patient demographic and physiological parameters at hospital admission and the risk of vascular occlusive events. To assess the extent to which risk of vascular occlusive events varies with severity of bleeding, we constructed a prognostic model for the risk of death due to bleeding and assessed the relationship between risk of death due to bleeding and risk of vascular occlusive events. There were 20,127 trauma patients with outcome data including 204 (1.01%) patients with a venous event (pulmonary embolism or deep vein thrombosis) and 200 (0.99%) with an arterial event (myocardial infarction or stroke). There were 81 deaths due to vascular occlusive events. Increasing age, decreasing systolic blood pressure, increased respiratory rates, longer central capillary refill times, higher heart rates and lower Glasgow Coma Scores (all p<0.02) were strong risk factors for venous and arterial vascular occlusive events. Patients with more severe bleeding as assessed by predicted risk of haemorrhage death had a greatly increased risk for all types of vascular occlusive event (all p<0.001).

**Conclusions:**

Patients with severe traumatic bleeding are at greatly increased risk of venous and arterial vascular occlusive events. Older age and blunt trauma are also risk factors for vascular occlusive events. Effective treatment of bleeding may reduce venous and arterial vascular occlusive complications in trauma patients.

## Introduction

Venous thromboembolic (VTE) events are an important cause of mortality and morbidity in trauma patients [2]–[4]. The spectrum of disease varies from occult deep vein thrombosis (DVT) to rapidly fatal pulmonary embolism (PE). Although the primary rationale for the prevention and treatment of DVT is to reduce the risk of PE, DVT itself can cause serious morbidity, including permanent deep venous insufficiency, chronic post-thrombotic phlebitis, prolonged hospital stay and delayed rehabilitation [5]. Although the prevention of VTE events is an important part of effective trauma care, there is little reliable information on risk factors for VTE in trauma patients.

With the increase in the average age of trauma patients in many high income countries [6] there is growing concern about the risk of arterial vascular occlusive events following trauma. The tachycardia induced by pain and haemorrhage increases myocardial oxygen demand whilst blood loss reduces haemoglobin concentration and myocardial oxygen delivery. Among patients in whom myocardial oxygen extraction is near maximal at rest, trauma could precipitate myocardial ischemia. Although cardiovascular events are likely to become an increasingly important factor complicating recovery following trauma, once again, little is known about the risk factors for arterial events in trauma patients.

We used data from the CRASH-2 trial to identify risk factors for venous and arterial vascular occlusive events in a large international cohort of trauma patients. The CRASH-2 trial included 20,211 bleeding trauma patients from 270 hospitals in 40 countries and because it has almost complete data at hospital admission and follow-up it provides a unique resource to examine risk factors for vascular occlusive events following trauma.

## Methods

### Sample

We examined data from the CRASH-2 trial, a large international, double-blind, randomised, placebo-controlled trial of the effects of tranexamic acid, on death and vascular occlusive events in bleeding trauma patients. Detailed information on the methods of the CRASH-2 trial have been published previously [1]. Overall 20,211 patients were randomised to receive tranexamic acid or placebo with 99.6% follow-up.

### Outcomes

Outcomes were fatal and non-fatal vascular occlusive events including deep vein thrombosis, pulmonary embolism, myocardial infarction and stroke. To assess the extent to which factors predictive of death due to bleeding also predicted the risk of vascular occlusive events, death due to bleeding was also examined as an outcome.

### Risk factors

Demographic, injury and patient physiological data obtained prior to randomisation were used as potential risk factors that might predict vascular occlusive events or death due to bleeding.

### Statistical analysis

FMeans and standard deviations were calculated for continuous variables such as age and physiological parameters. Risk factors were categorised prior to analysis. Potential continuous physiological risk factors were categorised using clinically relevant boundaries, combining categories where there were too few events for meaningful analysis. Country of randomisation was categorised as low, middle or high income according to the World Bank categorisation [7]. A complete case analysis was carried out since there were very few missing data. Univariate analyses were performed using the χ^2^ method. Multivariate analysis for each of the vascular occlusive outcomes and death due to bleeding were performed using a conceptual framework delineating distal from proximal risk factors to avoid inappropriately adjusting for mediating factors [8] (conceptual framework available on request). Odds ratios and 95% confidence intervals were estimated. Where appropriate, tests for trend and departures from linearity were conducted using likelihood ratio tests.

A model predicting risk of death due to bleeding was created using a backwards stepwise logistic regression starting with the inclusion of all baseline variables that had been studied in univariate analysis. The model contained an interaction term between time since injury and treatment group as there had been strong evidence for effect modification between these two variables in previously published CRASH-2 analyses [9]. The final model was used to construct tertiles of probability of death due to bleeding. We compared vascular occlusive event rates across predicted tertiles of death due to bleeding using χ^2^ methods and score tests for trend where appropriate. Analysis was performed in Stata (StataCorp. 2011. *Stata Statistical Software: Release 12*. College Station, TX: StataCorp LP). All CRASH-2 collaborators obtained local ethics and research committee approval.

## Results

From the initial 20,211 randomised, 20,127 patients completed follow-up (99.6%). The majority of trauma patients in this study were from low-middle income countries (97.9%) and were male (83.8%). More than two thirds of patients were seen within 3 hours of injury (67.0%) and most had sustained blunt injury, either singly or in combination with penetrating injury (67.6%).

The risk of venous occlusive events within this cohort was 1.01% with 204 patients having either a PE, DVT or both. Within this group 123 (60.3%) patients were diagnosed with PE, 61 (29.9%) with DVT and 20 (9.8%) patients with both. There were 200 (0.99%) arterial occlusive events. There were 77 (38.5%) patients with MI, 110 (55%) with stroke and 13 patients (6.5%) with both MI and stroke. Overall, 3,076 patients died following trauma, 81 (2.6%) from vascular occlusive events, including 29 from MI, 13 from stroke and 39 from PE. There were 1,063 (34.6%) deaths due to bleeding.

Univariate analysis showed strong evidence (all p<0.001) of an association between several potential risk factors and vascular occlusive events and death due to bleeding (see Table 1). In multivariate models (see Table 2), increasing age, decreasing systolic blood pressure, increased respiratory rates, prolonged central capillary refill times, higher heart rates and lower Glasgow Coma Score (GCS) predicted all three outcomes of venous and arterial occlusive events and death due to bleeding (all p<0.02). In addition venous and arterial occlusive events were associated with blunt injury, whereas death due to bleeding was associated with penetrating injury (all p<0.05).

**Table 1:** Univariate analysis* of 20,127 trauma patients with outcome data from the CRASH2 trial, showing odds ratios (95% CI) for risk factors for fatal and non-fatal venous (PE and VT), arterial (MI and stroke) and death due to bleeding.

	Totals	Venous events (%)	p value	Arterial events (%)	p value	Death due to bleeding (%)	p value
**Sex**							
Male	16,869	158 (0.94)	p = 0.013	161 (0.95)	p = 0.201	884 (5.24%)	p = 0.551
Female	3,257	46 (1.41)		39 (1.20)		179 (5.50%)	
Not known	1						
**Age (years)**							
<25	5,615	42 (0.75)	p<0.001	32 (0.57)	p<0.001	260 (4.63)	p = 0.002
25-<35	6,076	56 (0.92)		38 (0.63)		325 (5.35)	
35-<45	3,798	40 (1.05)		33 (0.87)		197 (5.19)	
45-<55	2,471	31 (1.25)		34 (1.38)		146 (5.91)	
> = 55	2,166	35 (1.62)		63 (2.91)		135 (6.23)	
Not known	1						
**Time since injury (hours)**							
< = 1	7,451	62 (0.83)	p = 0.138	76 (1.02)	p = 0.431	484 (6.50)	p<0.001
>1-< = 3	6,033	69 (1.14)		52 (0.86)		331 (5.49)	
>3	6,634	73 (1.10)		72 (1.09)		247 (5.28)	
Not known	9						
**Type of injury**							
Penetrating only	6,522	164 (1.21)	p<0.001	41 (0.63)	p<0.001	408 (6.26)	p<0.001
Blunt and penetrating	13,605	40 (0.61)		159 (1.17)		655 (4.81)	
**SBP (mmHg)**							
<80	3,305	55 (1.66)	P<0.001	51 (1.54)	P<0.001	543 (16.43)	P<0.001
80-<90	3,154	58 (1.84)		50 (1.59)		209 (6.63)	
90-<100	4,151	34 (0.82)		33 (0.79)		160 (3.85)	
100-<120	5,180	28 (0.73)		29 (0.56)		98 (1.89)	
> = 120	4,308	19 (0.44)		37 (0.86)		51 (1.18)	
Not known	29						
**RR (per min)**							
0-<20	4,845	25 (0.52)	p<0.001	46 (0.95)	p<0.001	220 (4.54)	p<0.001
20-<25	8,482	69 (0.81)		50 (0.59)		285 (3.36)	
> = 25	6,614	110 (1.66)		102 (1.54)		504 (7.62)	
Not known	186						
**CCRT (secs)**							
< = 2	6,809	40 (0.59)	p<0.001	28 (0.41)	p<0.001	183 (2.69)	p<0.001
3-< = 4	9,357	85 (0.91)		95 (1.02)		508 (5.43)	
>4	3,354	71 (2.12)		66 (1.97)		350 (10.44)	
Not known	607						
**HR (bpm)**							
0-< = 90	2,768	19 (0.69)	p<0.001	32 (1.16)	p<0.001	196 (3.77)	p<0.001
90-< = 100	6,368	33 (0.52)		35 (0.55)		125 (3.17)	
100-< = 110	3,712	33 (0.89)		34 (0.92)		148 (3.99)	
110-< = 120	3,656	41 (1.12)		33 (0.90)		217 (5.94)	
>120	3,486	78 (2.24)		65 (1.86)		326 (9.35)	
Not known	137						
**GCS (total)**							
Mild	13,792	104 (0.75)	p<0.001	60 (0.44)	p<0.001	493 (3.57)	p<0.001
Moderate	2,693	43 (1.60)		55 (2.04)		214 (7.95)	
Severe	3,619	57 (1.58)		85 (2.35)		354 (9.78)	
Not known	23						

p values are likelihood ratio tests for association.

* univariate analysis with χ2 test.

SBP: systolic blood pressure; RR: respiratory rate; CCRT: capillary refill time; HR: heart rate in beats per minute; GCS: Glasgow Coma Score; mild (13–15), moderate (9–12), severe (3–8).

**Table 2:** Multivariate adjusted odds ratios (95% confidence intervals) for demographic and physiological risk factors for fatal and non-fatal venous (DVT and PE) and arterial events (MI and stroke) and death due to bleeding for 20,127 adult trauma patients from the CRASH2 trial.

		Venous events		p value		Arterial events		p value		Death due to bleeding	p value
**Sex**											
Male		1.00				1.00				1.00	
Female		1.29 (0.92–1.81)		0.142^A^		0.94 (0.66–1.35)		0.730^A^		1.07 (0.90–1.26)	0.461^A^
**Age (years)**											
<25		1.00				1.00				1.00	
25-<35		1.20 (0.80–1.80)				1.08 (0.67–1.73)				1.17 (0.99–1.39)	
35-<45		1.34 (0.87–2.08)				1.50 (0.92–2.44)				1.15 (0.95–1.40)	
45-<55		1.48 (0.93–2.37)				2.28 (1.40–3.71)				1.34 (1.09–1.66)	
> = 55		1.69 (1.06–2.68)		0.017* A		4.63 (2.99–7.18)		<0.001* A		1.44 (1.16–1.80)	<0.001* A
**Type of injury**											
Penetrating only		1.00				1.00				1.00	
Blunt and penetrating		1.66 (1.16–2.37)		0.004^A^		1.44 (1.01–2.05)		0.039^A^		0.71 (0.62–0.81)	<0.001^A^
**Time since injury (hours)**											
< = 1		1.00				1.00				1.00	
>1-< = 3		1.19 (0.84–1.70)				0.72 (0.51–1.04)				0.84 (0.73–0.98)	
>3		1.10 (0.78–1.56)		0.602^B^		0.86 (0.62–1.21)		0.213^B^		0.57 (0.49–0.67)	<0.001^B^
**SBP (mmHg)**											
<80		3.67 (2.15–6.24)				1.45 (0.94–2.26)				13.68 (10.19–18.35)	
80-<90		4.20 (2.49–7.08)				1.83 (1.18–2.82)				5.79 (4.24–7.90)	
90-<100		2.07 (1.17–3.64)				1.12 (0.69–1.80)				3.55 (2.58–4.88)	
100-<120		1.88 (1.08–3.28)		<0.001* C		0.84 (0.51–1.37)		0.004* C		1.74 (1.23–2.45)	<0.001* C
> = 120		1.00				1.00				1.00	
**RR (per min)**											
0-<20		1.00				1.00				1.00	
20-<25		1.92 (1.21–3.07)				0.80 (0.53–1.21)				0.84 (0.70–1.00)	
> = 25		3.68 (2.37–5.72)		<0.001* D		1.82 (1.27–2.60)		<0.001^D^		1.80 (1.52–2.12)	<0.001^D^
**CCRT (secs)**											
< = 2	1.00				1.00				1.00		
3-< = 4	1.46 (1.00–2.13)				1.99 (1.30–3.05)				1.89 (1.59–2.25)		
>4	3.02 (2.03–4.51)		<0.001* E		3.27 (2.07–5.15)		<0.001* E		3.51 (2.91–4.24)		<0.001* E
**HR (bpm)**											
0-< = 90	1.38 (0.77–2.44)				1.57 (0.92–2.66)				1.13 (0.90–1.42)		
90-< = 100	1.00				1.00				1.00		
100-< = 110	1.84 (1.03–3.28)				1.66 (0.95–2.89)				1.22 (0.96–1.56)		
110-< = 120	2.27 (1.30–3.96)				1.54 (0.88–2.71)				1.82 (1.45–2.28)		
>120	4.04 (2.39–6.82)		<0.001^F^		2.55 (1.53–4.27)		<0.001^F^		2.65 (2.13–3.29)		<0.001^F^
**GCS (total score)**											
Mild (13–15)	1.00				1.00				1.00		
Moderate (9–12)	1.74 (1.21–2.51)				4.26 (2.92–6.22)				1.71 (1.44–2.04)		
Severe (3–8)	1.65 (1.18–2.32)		0.002^C^		4.92 (3.47–7.00)		<0.001^C^		2.49 (2.13–2.90)		<0.001* C

p values are likelihood ratio tests for association.

* likelihood ratio test for trend.

SBP: systolic blood pressure; RR: respiratory rate; CCRT: capillary refill time; HR: heart rate in beats per minute; GCS: Glasgow Coma Score.

A : Model 1 includes country, sex, age and type of injury.

B : Model 2 includes model 1 and time since injury.

C : Model 3 includes model 2 and SBP and GCS.

D : Model 4 includes model 2 and RR and GCS.

E : Model 5 includes model 2 and CCRT and GCS.

F : Model 6 includes model 2 and HR and GCS.

**Figure 1:**
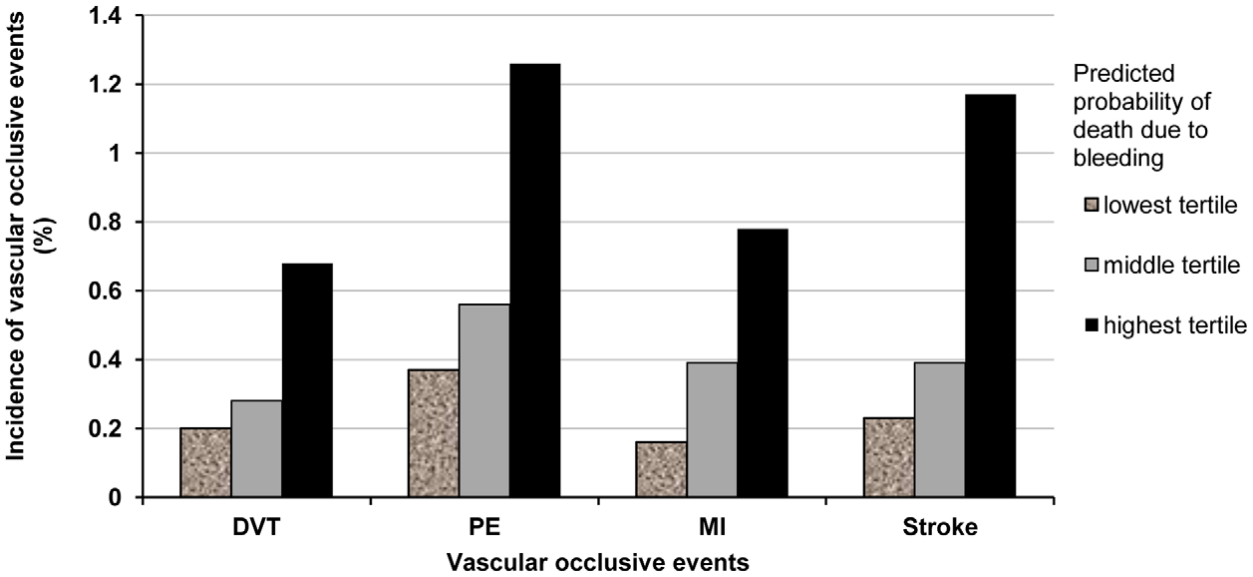
Incidence of vascular occlusive events by tertiles of predicted probability of death due to bleeding for 19,298 adult trauma patients without missing data.

The analysis of vascular occlusive events across tertiles of predicted death due to bleeding (see Figure 1) showed increasing risk of all categories of vascular occlusive events with increasing probability of death due to bleeding (score test for trend p<0.001 for all vascular occlusive events).

## Discussion

### Principle findings

Venous and arterial occlusive events following trauma share many risk factors including increasing age, blunt injury and worsening physiological parameters. The risk of all types of vascular occlusive events was greatest in patients at highest risk from death due to bleeding.

### Strengths and limitations of the study

We examined clinically relevant demographic and physiological parameters as risk factors for vascular occlusive events. These factors can all be easily measured in the initial assessment of a trauma patient. In the CRASH-2 trial, they were measured and recorded in a standardised way before the outcome was known thus reducing the potential for diagnostic bias. Because of the large sample size with minimal missing data our estimates are considerably more precise than those of previous studies. The use of a large international trauma population increases the extent to which our results can be generalised.

Use of thromboprophylaxis was not recorded in the CRASH-2 trial and may have been an important confounder of the association between age, type of injury and physiological risk factors for vascular occlusive events. It is hard to predict the direction of any confounding effect from thromboprophylaxis. It might have been that treating clinicians withheld thromboprophylaxis from patients considered at greatest risk from their bleeding as measured by poor physiological parameters including low GCS scores and this could have contributed to a greater number of venous occlusive events in these groups. The presence of head injury was not measured on enrolment forms but later as part of outcome collection thus was not used in this cohort analysis to avoid observer bias. Head injury may have confounded the relationship between GCS and our outcome measures and inclusion in our analysis would have helped us study the role of GCS as a marker of shock separate to head injury.

As in all studies, there would have been some misclassification of both exposures and outcomes. Non-differential exposure misclassification tends on average to reduce measured associations between exposure and outcomes biasing results towards the null, although this cannot be guaranteed for any one particular study. Participating centres were asked to exercise high specificity when recording outcome data to reduce any bias but the diagnosis of vascular occlusive events may have been differential between suspected at-risk groups such as the elderly or the more severely injured which might have affected our effect measures. Despite the request for high specificity in recording outcome measures the mode of investigation and diagnostic parameters were not pre-specified thus there may be heterogeneity within outcome measurement which could weaken the association seen with putative risk factors.

### Risk factors compared with other literature

Few previous studies have investigated the association between physiological parameters and the risk of venous occlusive events in trauma patients. Knudson et al [4] found no independent association between hypotension at admission and risk of DVT or PE. However, this study adjusted for major operative procedures and ventilator days which may be on the causal pathway between shock on admission and the outcome of interest. Napolitana et al [10] found that Revised Trauma Scores that include a combination of GCS and respiratory rate were associated with an increased risk of DVT. Other studies have also found an association between low GCS scores and risk of venous occlusive events [11], [12] in trauma patients. Lower GCS scores could represent more severe head injury and this has been shown to be an independent risk factor for the development of venous thrombosis [11], [13], [14]. Head injury patients may be more at risk of venous thrombosis from prolonged immobilisation and delayed deployment of thromboprophylaxis [15]–[17].

Our study shows increasing age to be a strong predictor of both venous and arterial occlusive events. Other studies have also found older age to be a predictor of DVT and PE [10], [12], [13], [18], [19]. We are not aware of any previous literature on the association between age and arterial events within the trauma population. Only two other studies have looked at myocardial infarction in trauma patients and these reported incidence rates only and were not able to study risk factors [20], [21]. However, the association between increasing age and arterial occlusive events in our study agrees with the large body of evidence describing increasing age as a predictor of cardiovascular disease in the general population [22], [23]. With increasing age there is an accumulation of atherosclerotic disease, and rising incidence of hypertension [24] and hypercholesterolaemia [25].

We have shown that the physiological risk factors that predict venous and arterial events within our trauma cohort also predict risk of death due to bleeding and that the risk of all vascular occlusive events increases with predicted risk of death due to bleeding. Physiological parameters including respiratory rate, heart rate, systolic blood pressure and level of consciousness are used in the assessment of blood loss and classification of shock within the ATLS guidelines which are used widely in the treatment of trauma patients [26]. Increased central capillary refill time as a measure of decreased peripheral perfusion has also been correlated with circulatory shock and poor outcomes in trauma patients [27], [28]. These initial physiological measures are surrogate markers of blood loss from trauma and those with greatest blood loss would appear to be at greatest risk for both venous and arterial occlusive events in our study.

### Possible mechanisms

The severity of bleeding was strongly related to the risk of vascular occlusive events. As regards myocardial infarction, it is possible that this could be a direct causal relationship whereby bleeding compounds any imbalance in myocardial oxygen supply and demand thus precipitating myocardial ischemia. Indeed, this may explain the observation from the CRASH-2 trial that tranexamic acid administration reduced the risk of myocardial infarction through the proposed mechanism of reducing bleeding [1]. Trauma may illicit some of the same stressors as surgery and studies investigating perioperative myocardial infarction describe myocardial oxygen supply-demand imbalance from hypotension and postoperative tachycardia, as a cause of MI in surgical patients [29], [30].

On the other hand severe trauma through tissue injury, initiates the systemic inflammatory response syndrome (SIRS), characterised by up-regulation of the innate immune response with systemic activation and release of pro-inflammatory cytokines, chemokines, free radical products and “damage”-associated molecular patterns (DAMPs) [31]–[34]. Inflammation has a well recognised role in the pathogenesis of acute coronary syndromes, contributing to local plaque rupture and thrombosis within the coronary circulation [35]–[37]. Thus the extent of bleeding may simply be a marker of the severity of tissue damage and the resulting inflammatory response. Pro-inflammatory mechanisms may also explain why blunt versus penetrating trauma was associated with arterial events in our study as we can hypothesise that blunt trauma produces greater and more distributed tissue injury thus providing a stronger inflammatory response. Severity of injury has been associated with pro-inflammatory cytokine levels including IL-6 and IL-10 [38], [39]. The interruption of inflammatory pathways may provide another role for tranexamic acid in reducing the risk of myocardial infarction in this setting. Tranexamic acid prevents the binding of plasminogen to fibrin which activates plasmin. Plasmin is not only involved in fibrinolysis but also activates many key inflammatory components including neutrophils and macrophages and is a potent chemoattractant for immune cells [40].

The possible mechanisms underlying venous vascular events after trauma have previously been described by Virchow's triad of stasis, hypercoagulability and endothelial cell damage [41]. Previous studies have demonstrated a hypercoagulable state days after the initial injury [42], [43]. Inflammatory processes may also be playing a role in the development of venous occlusive events as there is considerable cross-talk between the coagulation and inflammation pathways [44]. In addition to the initial injury, the sickest patients will undergo multiple procedures and have prolonged hospital admission both of which can contribute to increased risk for venous occlusive events [45]. Patients with severe and ongoing haemorrhage may have been deemed to be too high risk to receive thromboprophylaxis which could result in increased venous occlusive events in these patients.

### Implications of the study

Our study highlights the importance of both venous and arterial vascular events in the bleeding trauma patient. Although clinicians treating trauma patients have long prioritised stopping bleeding and preventing venous occlusive complications, clinicians should also be aware of arterial complications. Another implication of our study is that measures taken to reduce bleeding in trauma patients might in turn reduce the risk of vascular occlusive complications.
